# Ultrasound tomography enhancement by signal feature extraction with modular machine learning method

**DOI:** 10.1371/journal.pone.0297496

**Published:** 2024-01-31

**Authors:** Bartłomiej Baran, Dariusz Majerek, Piotr Szyszka, Dariusz Wójcik, Tomasz Rymarczyk

**Affiliations:** 1 Research & Development Centre Netrix S.A., Lublin, Poland; 2 Faculty of Fundamentals of Technology, Lublin University of Technology, Lublin, Poland; 3 WSEI University, Lublin, Poland; Islamia University of Bahawalpur: The Islamia University of Bahawalpur Pakistan, PAKISTAN

## Abstract

Robust and reliable diagnostic methods are desired in various types of industries. This article presents a novel approach to object detection in industrial or general ultrasound tomography. The key idea is to analyze the time-dependent ultrasonic signal recorded by three independent transducers of an experimental system. It focuses on finding common or related characteristics of these signals using custom-designed deep neural network models. In principle, models use convolution layers to extract common features of signals, which are passed to dense layers responsible for predicting the number of objects or their locations and sizes. Predicting the number and properties of objects are characterized by a high value of the coefficient of determination *R*^2^ = 99.8% and *R*^2^ = 98.4%, respectively. The proposed solution can result in a reliable and low-cost method of object detection for various industry sectors.

## Introduction

The nature of waves implies that their propagation, along with accompanying phenomena such as interference or absorption, has to be described by differential equations. They allow us to consider various properties of the environment that affect wave propagation. In the case of ultrasound waves, which can be generated by transducers made of piezoelectric materials [1, 2], phenomena, such as sound pressure, energy transfer, material acoustic impedance, reflection, and interference effects must be taken into account. In the time-dependent domain, the appropriate method to numerically simulate these phenomena is a set of differential equations that can be solved by a finite element method with the appropriate boundary conditions. This set of equations is called a convected wave equation (CWE) model, whose derivation starts with continuity, momentum, and equation of state as follows:

$$\begin{array}{c} \frac{\partial \rho }{\partial t}+\nabla \cdot \left(\rho u\right)=0, \end{array}$$ (1)

$$\begin{array}{c} \frac{\partial \rho u}{\partial t}+\nabla \cdot \left(pI+\rho uu^{T}\right)=0, \end{array}$$ (2)

$$\begin{array}{c} \rho =\rho (p), \end{array}$$ (3)

where **I** denotes the identity matrix, *ρ* the density of the medium, *p* pressure, and **u** represents a vector of medium velocities *u*. Next, the above equations are linearized using the following expressions:

$$\begin{array}{c} \rho =\rho_{0}+\rho_{\Delta }\left(x,t\right), \end{array}$$ (4)

$$\begin{array}{c} u=u_{0}+u_{\Delta }\left(x,t\right), \end{array}$$ (5)

$$\begin{array}{c} p=p_{0}+p_{\Delta }\left(x,t\right), \end{array}$$ (6)

where *p*_Δ_, *ρ*_Δ_, and *u*_Δ_ represent an evolution (in space *x* and time *t*) of values of pressure, density, and flow velocity, while *p*_0_, *ρ*_0_, and *u*_0_ denote their background values. Substituting Eqs 6, 5 and 4 into 1, 2, 3 gives a linearized form of the continuity, momentum, and equation of state:

$$\begin{array}{c} \frac{\partial \rho_{\Delta }}{\partial t}+\nabla \cdot \left(\rho_{\Delta }u_{0}+\rho_{0}u_{\Delta }\right)=0, \end{array}$$ (7)

$$\begin{array}{c} \rho_{0}\frac{u_{\Delta }}{\partial t}+u_{0}\frac{\rho_{\Delta }}{\partial t}+\nabla \cdot \left(p_{\Delta }I+\rho_{\Delta }u_{0}u_{0}^{T}+\rho_{0}u_{\Delta }u_{0}^{T}+\rho_{0}u_{0}u_{\Delta }^{T}\right)=0, \end{array}$$ (8)

$$\begin{array}{c} \rho_{\Delta }=\frac{\rho (p)}{c_{0}^{2}}, \end{array}$$ (9)

where *c*_0_ denotes the speed of sound and all second-order terms such as ∇ ⋅ *ρ*_Δ_**u**_**Δ**_ are omitted because the emphasis is on acoustic disturbances.

Originally, the CWE set was derived by Pierce assuming an adiabatic equation of state in the presence of a background flow [3, 4]. This mathematical approach allows for time-dependent simulation of complicated problems, resulting in the spatial distribution of density or medium velocity. In addition, it allows for simulation of the experimental setup revealing its properties and behavior of ultrasound waves. What is more important, it gives access to the imitation of the single ultrasonic transducer and the time-dependent sound pressure it records. This signal contains information about the processes that occurred during propagation through a studied measurement system.

Although wave propagation is a complex problem, this paper presents an approach to analyzing time-dependent ultrasonic signals that provide information about inclusions responsible for wave propagation disturbances. The article does not focus on solving the differential equations. Instead, investigation is based on real measurement data obtained by ultrasound tomograph and artificial intelligence algorithms. The key idea is the simultaneous analysis of signals recorded by three transducers. Using the prepared dataset, one is able to train deep neural network models for object detection that can predict the number of inclusions in the experimental setup or their coordinates and diameters. Utilizing measurement data from a small number of sensors allows for high time resolution of predictions, on the order of milliseconds. Nowadays, ultrasonic measurement methods are well developed and give great results, e.g. in medicine or industry, where ultrasonic probes with multiple signal transducers [5–10]. These imaging methods require large amounts of data to be processed. This is reasonable to obtain high-quality images. However, for industrial purposes, where the main goal e.g. is to detect single inclusions in a homogeneous substrate, the use of such a large amount of data is less reasonable.

The solution proposed in this article may be classified for the group of measurement methods of industrial process tomography (IPT). IPT is widely used in the monitoring and visualization of closed industrial pipelines and industrial reactors. It is mainly applied in the refining, food, and pharmaceutical industries and in municipal service facilities such as sewage treatment plants [11–15]. IPT deals with very complex problems aimed at monitoring and then optimizing technological processes using various measurement methods, including X-ray diffraction tomography [16] or microtomography [17], electrical resistance [18] and electrical capacitance tomography [19]. The advantage of using ultrasonic methods [20, 21] over other methods is that they provide a higher level of operational safety than X-ray methods, they can be used in areas without restrictions, and the results are easy to interpret.

In this article, several significant contributions are presented to the field of ultrasound tomography enhancement through signal feature extraction and the application of modular machine learning methods. A novel approach to object detection in the domain of ultrasound tomography is introduced, where time-dependent ultrasonic signals are analyzed through machine learning techniques, allowing for the identification of common features and the prediction of object characteristics within the imaging area. The core of the contribution is found in the custom-designed deep neural network models, where convolution layers are combined with dense layers for precise predictions. These models are distinguished by its exceptional performance, as evidenced by the outstanding results achieved using selected score functions. Furthermore, the potential of signal analysis from a limited number of measurement channels is underscored as a reliable and cost-effective method for object detection across various applications, including industrial and medical settings. This approach not only simplifies the procedure of object detection but also offers high time resolution for predictions, with measurement and prediction times on the order of milliseconds.

The paper is organized as follows. In the section Materials and Methods, we present a data acquisition procedure and design of deep learning models. Our main results and discussion are presented in the section Results and Discussion. Section Conclusion summarizes the major conclusions and gives a brief outlook of the limitations of the proposed work.

## Materials and methods

### Measurement data acquisition

**Fig 1:**
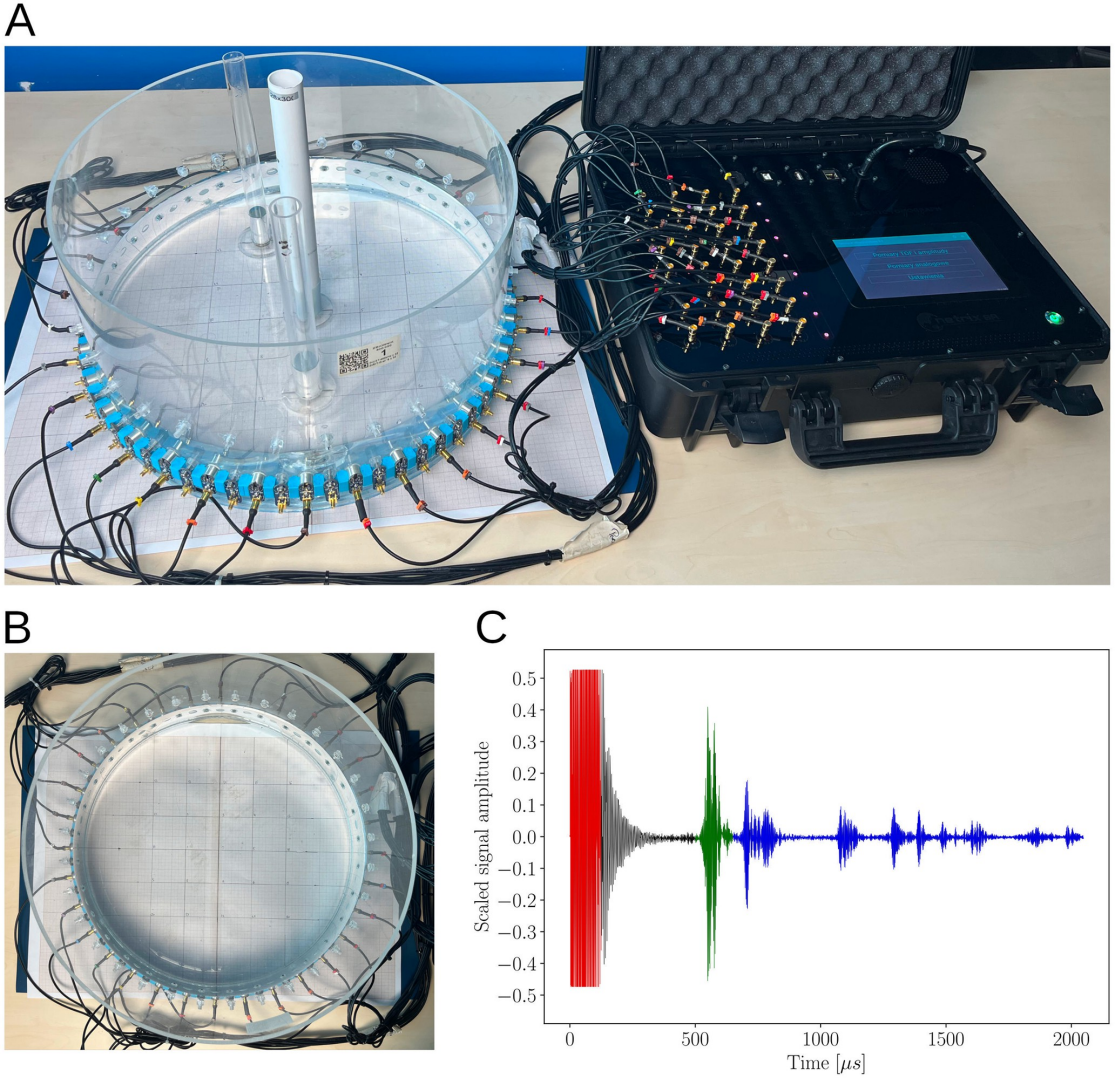
The experimental setup and example of measurement data: a) picture of a complete experimental setup, b) an experimental tank equipped with 32 ultrasound sensors arranged clockwise, c) an example of recorded data from a channel number 1.

Before presenting the main idea of the research method, i.e. how the recorded acoustic pressure caused by the propagation of ultrasonic waves allows machine learning algorithms to detect objects, it is necessary to briefly introduce the procedure of signal generation and data acquisition. Fig 1A shows a measurement system consisting of an ultrasonic tomograph and measurement tank. The tank, see Fig 1B, is filled with water and equipped with 32 ultrasonic transducers arranged clockwise, which are connected to the main unit responsible for controlling a measurement sequence. The ultrasound tomograph allows for any signal generation procedure and data reading from selected sensors, resulting in the possibility of the desired arrangement of measurement sequence [21]. In this case, the device generates an excitation signal consisting of 3 pulses and records the response for a single sensor, this is repeated for all other probes.

Fig 1C shows an example of the recorded signal from a single transducer for a tank without any inclusion. The individual areas corresponding to excitation (red), reflection from the opposite wall of the tank (green), and interference of reflected waves (blue) can be detected.

Single case of measurement is when the phantoms are placed in the tank, and their coordinates and diameter are recorded. The center of the coordinate system is at the center of the tank, where sensor 1 is on the Y-axis below the center. The measurements were carried out for ultrasonic frequency of 400kHz, with 8192 samples collected at 4MHz sampling rate and a signal amplification of 32dB with an analog converter offset equal to half of its range. The dataset includes 50 different measurements for a different number of objects inside the tank, from single to quadruple inclusions, with three replicates of each. The measurements were collected three times to ensure that the noise spectrum from the experimental system is captured, which is important during the model training. The experiment used inclusions of three different sizes and also collected measurements for an empty tank.

In the next step, data preparation is performed by merging signals from three neighboring channels separated by a central angle equal to *ϕ* = 2*πn*/32, where *n* denotes an integer that is the absolute value of the difference in the ordinal number of sensors between adjacent channels. For each set of sensors, it is necessary to select a new coordinate system, which is obtained by rotating the initial coordinate system. This transformation is performed relative to the center of the tank by an angle equal to −2*π*(*i* − 1)/32, where *i* = 1, 2, 3… denotes the number of the central channel in the considered triple. It allows to determine the positions of objects in the new coordinate system. The result of such an operation is a dataset of 24,000 different data frames, which is available online [22]. An example of the single data frame is presented in Fig 3.

### Model architecture

This section introduces the modular machine learning models, their main concepts, and their architecture.

There a two predictive models used in the study. One is a classification model, which is used to determine the number of inclusions in a tank. The second model is a regression model to return the positions and diameters of inclusions.

The key idea is to combine a block consisting of convolution layers with a block of dense layers. The crucial task was to recognize patterns in raw signals connected with inclusions. The use of convolution layers is dictated by the fact that the input to the model is a set of signals from three channels treated as an image of dimension 3 × 8192. The first convolutional layer (Conv2D) is used to extract key information from three channels simultaneously. The convolutional networks are inherently translation invariant, meaning they can recognize patterns regardless of their location in the input. This property is crucial for tasks like image classification [23, 24], where the position of objects may vary. The outputs of the first convolution layer are a kind of time series containing information from the three channels. Previous attempts to treat signals from these channels independently did not produce such good model-fitting results. The extracted information from the three channels is then processed by a one-dimensional convolution layer (Conv1D), which extracts information from the time series thus created. Conv1D works by applying a set of filters to the input sequence and sliding them across the data with a specified stride. Each filter performs a convolution operation, extracting local patterns and features from the input sequence. The resulting feature maps capture different aspects of the input data, such as local patterns, trends, or temporal dependencies. Convolution blocks exploit the spatial structure of the input data, reducing the overall computational burden compared to dense networks, which require connections between every pair of neurons.

The results from the convolution block are combined with dense layers, and then, depending on the tasks, the last layer of the network classifies the number of inclusions in the tank or predicts the location of these inclusions and their diameters. Dropout layers are added to avoid over-fitting.

Training of the model was done using the Nadam optimizer (which combines the benefits of Adam optimizer with Nesterov Accelerated Gradient, enhancing convergence during training by incorporating momentum-based updates and adaptive learning rate adjustments) and an initial learning rate equal to 0.001.

In the context of this research, it is essential to acknowledge that the determination of model architectures was achieved through a systematic trial and error approach. This approach encompassed a series of iterative experiments in which various architectural configurations were explored, performance was assessed, and refinements were made based on empirical results and insights derived from each trial.

**Fig 2:**
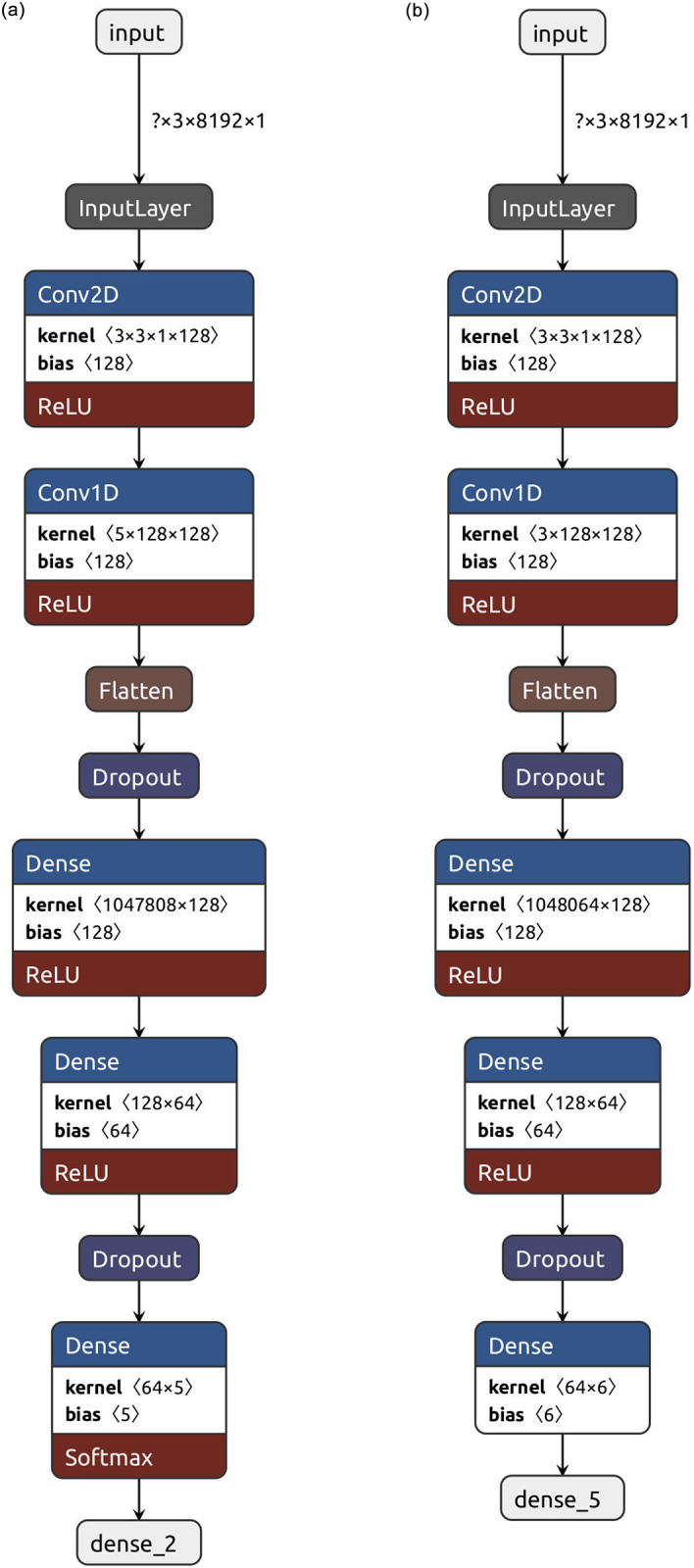
Architectures of both types of models used in the reconstruction. (a) The classification model (b) The regression model.

The extensive knowledge and experience of the research team are attributed to the success of our research in the domain of deep neural network modeling, with a particular focus on hyperparameter selection and architecture design. Expertise in these areas was employed to implement a systematic and effective approach for fine-tuning model hyperparameters and optimizing architectures, leading to the remarkable performance demonstrated in this study. A pivotal role in addressing the intricacies of ultrasound tomography and object detection using machine learning methodologies has been played by the accumulated know-how, which has been acquired through years of research and practical applications. The network architectures are similar and shown in Fig 2a and 2b.

Prediction of the location of inclusions and their diameters is carried out in two stages. The first is classification to indicate the number of inclusions in the tank. The second is the prediction of the position (*x*, *y*) and diameter of the inclusion *d*. However, it should be mentioned that for each number of inclusions (in the experiments the number of inclusions varied between 0 and 4) a separate regression model was used. Thus, the final reconstruction is the result of the prediction of both classification and regression models.

The models presented above (see Fig 2) were developed using the TensorFlow environment [25], which provides an advanced platform for building and training deep learning algorithms. The entire dataset was divided into training and testing subsets in a 9:1 ratio. This procedure enables to finding of optimal parameter values, providing at the same time, an objective evaluation of the quality of the fit. To monitor the training progress, a validation subset was extracted from the training set, constituting 10% of the training data. The validation set was used to track model performance during training and observe the occurrence of over-fitting. This approach ensures that model parameters are tuned based on a reliable measure of generalization performance, which ultimately leads to better model performance on unseen data. Before the classification model learning process, *one-hot* coding was applied to the categorical labels. The model learning process took 100 epochs, with each epoch involving a cycle of updating the model’s internal parameters by optimizing the categorical cross-entropy loss function defined as follows:

$$\begin{array}{c} L_{cross-entropy}=-\sum_{i=1}^{N}\sum_{j=1}^{M}\theta_{ij}\cdot \text{log}\left({\hat{\theta }}_{ij}\right), \end{array}$$ (10)

where *θ*_*ij*_ represents the true label for sample *i* and class *j*, which is 1 if the sample belongs to class *j* and 0 otherwise. ${\hat{\theta }}_{ij}$ denotes the predicted probability that sample *i* belongs to *j* calculated by soft-max function. To ensure a balanced and efficient training process, a batch-based approach was adopted, where each batch consisted of 32 samples. This facilitated the model’s effective gradient propagation and parameter adjustment during the training process.

Overall accuracy and confusion matrix were used to assess the fit of the classification model. Since the distribution of the dependent variable was balanced (each class occurred in the data set with the same size), the choice of accuracy as a measure of fit seems appropriate [26]. For regression models mean squared error (MSE) is defined as:

$$\begin{array}{c} MSE=\frac{1}{n}\sum_{i=1}^{n}{\left(\theta_{i}-\hat{\theta_{i}}\right)}^{2}, \end{array}$$ (11)

and

$$\begin{array}{c} R^{2}={\text{Cor}}^{2}\left(\theta ,\hat{\theta }\right), \end{array}$$ (12)

were used to assess fit, where *θ* and $\hat{\theta }$ mean observed and predicted inclusion coordinates, respectively. Moreover, MSE has been used in optimizing the loss function during the training of regression models.

## Results and discussion

**Fig 3:**
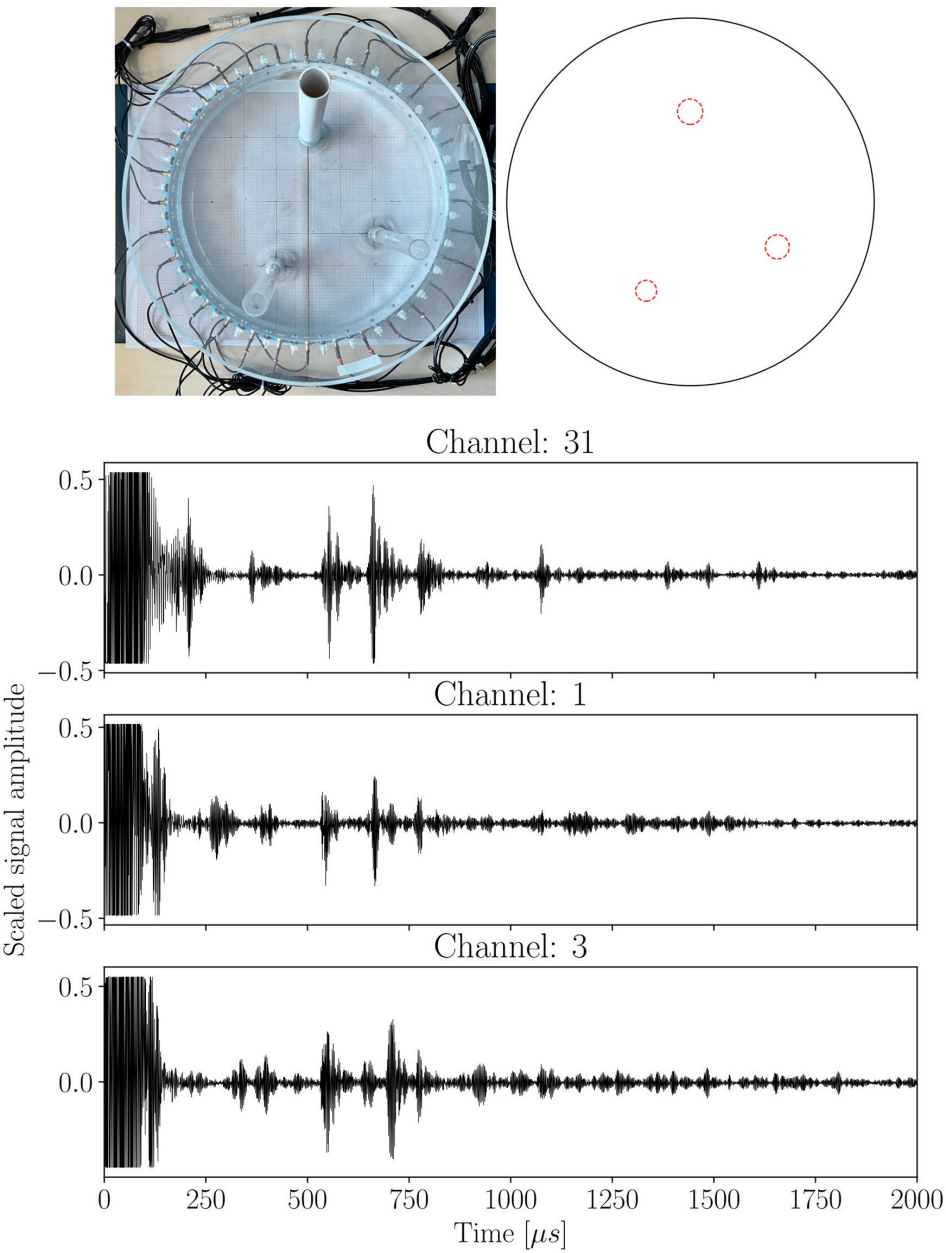
The example of a data frame with the corresponding prediction. Plot showing the scaled signals collected from three channels for a case with three inclusions and obtained prediction for this test data.

Fig 3 presents an example of a single data frame consisting of three measurements from neighboring channels with *ϕ* = *π*/8. The different shapes of each of the signals and positions of the peaks can be noticed, which is caused by the different distances of particular sensors from the inclusion. The magnitude of the peaks in the signals varies from each other significantly which is a consequence of the fact that every channel has its own sensitivity caused mainly by the quality of the sensor and other factors as sensor contact with the tank. Please note that here, the raw measured values from the transducer are not shown, but the normalized signal plot in the time domain shifted by its mean value. Moreover, Fig 3 shows an example of predicting this particular position of three inclusions and their diameters.

**Fig 4:**
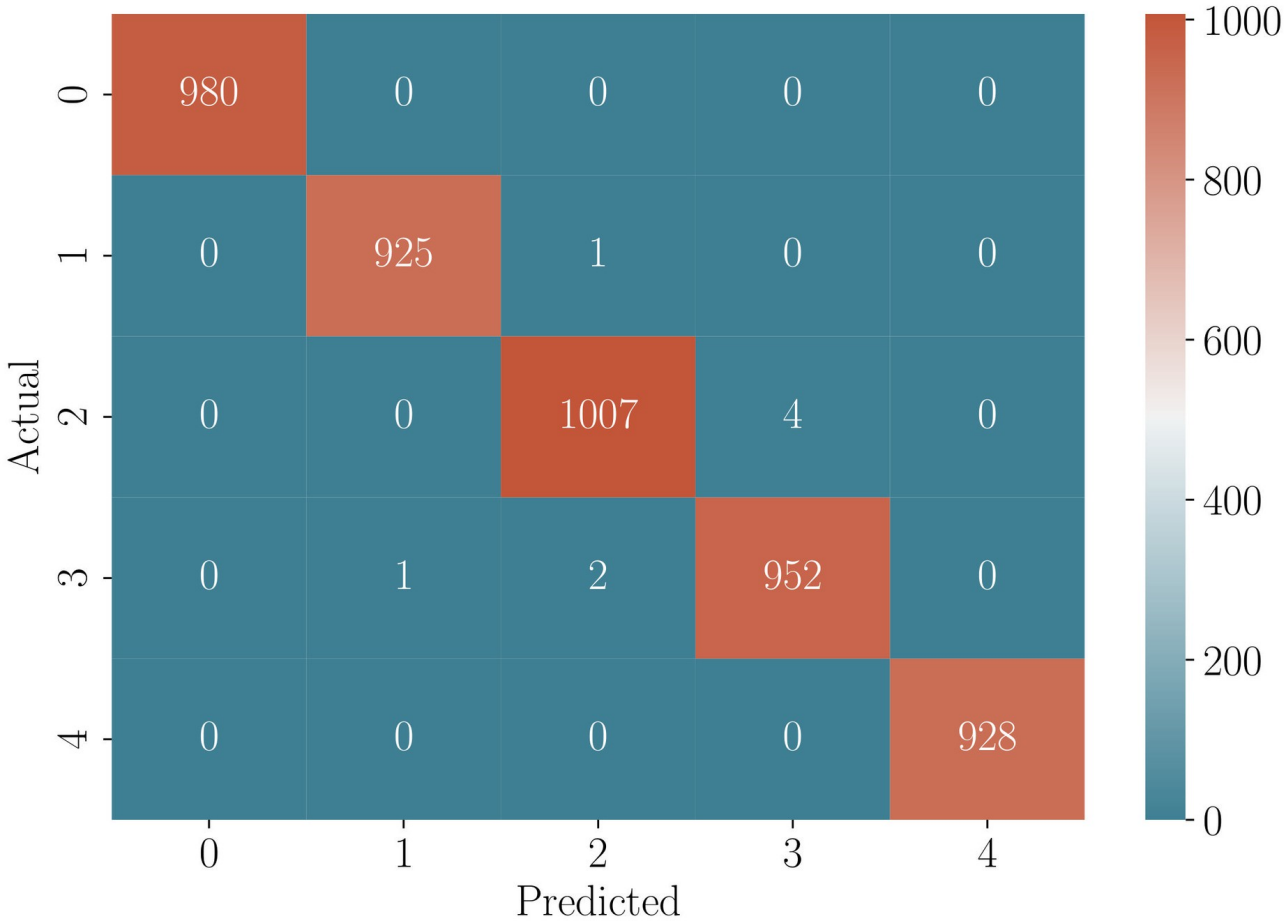
Confusion matrix. The values on the axes indicate the number of inclusions, predicted and observed, respectively.

The classification model has demonstrated an excellent fit, as indicated by the high accuracy level of 99.8% achieved on the test set. The confusion matrix revealed only 8 errors out of the 4800 observations in the test set (refer to Fig 4). This signifies the model’s strong ability to accurately classify instances. Similarly, the regression models exhibited a very high level of fit. The mean squared error (MSE) values ranged from 0.32 to 0.72, depending on the number of inclusions in the tank (see Table 1). These low MSE values indicate that the regression models have a small average deviation from the true values. Furthermore, the *R*^2^ measures, which assess the proportion of variance explained by the models ranged from 98.0% to 99.1%. These high *R*^2^ values indicate that the regression models capture a significant portion of the variation in the data.

**Table 1:** Measures of fit of regression models.

Number of inclusions	R^2^	MSE
1	99.1%	0.32
2	98.4%	0.59
3	98.0%	0.72
4	98.1%	0.67
**Average value**	98.4%	0.57

In conclusion, both the classification and regression models demonstrate a high level of fit, with accurate classification and low deviations in the regression predictions. These findings highlight the effectiveness and reliability of both types of models in capturing and explaining the underlying patterns in the data.

**Fig 5:**
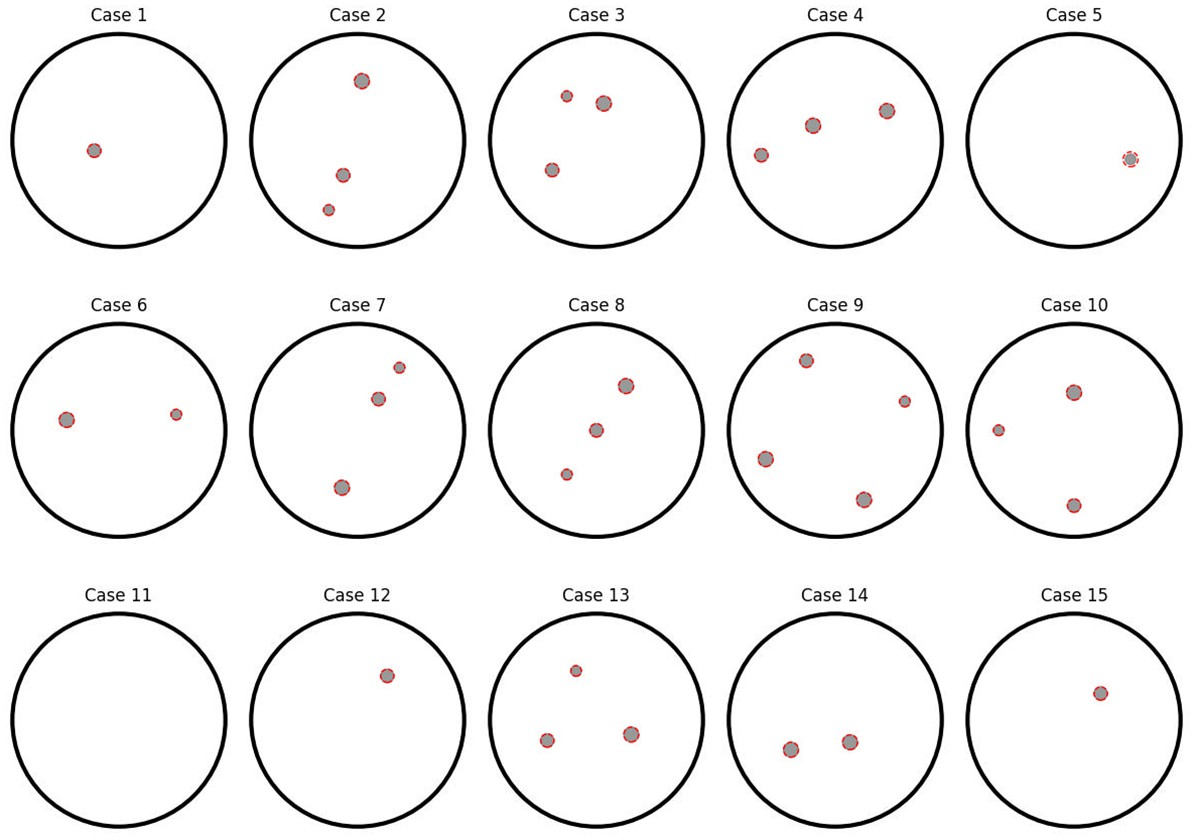
Examples of inclusion reconstruction for the test set. Solid gray circles indicate actual inclusions, while dashed red circles indicate positions and diameters predicted by the models.

The results of the reconstructions using the described models are presented in Fig 5. It is evident that the reconstructions exhibit a remarkably high level of accuracy, both in the absence of inclusions and for varying numbers of inclusions. This demonstrates the models’ ability to accurately predict and capture the underlying patterns in the test set.

In Fig 5, the gray circles represent the actual inclusions, while the red circles represent the predictions generated by the model. The close alignment between the actual and predicted inclusions further emphasizes the models’ effectiveness in capturing the true characteristics of the data.

The practical implications of our research are substantial. In the field of ultrasound tomography, where non-invasive imaging and object detection are essential, our approach offers a cost-effective and efficient solution. The ability to detect and locate objects within a medium, such as detecting tumors in medical imaging or defects in industrial materials, can significantly impact diagnostic and processes of quality control.

The reduction in the number of sensors required for this level of accuracy is a noteworthy advancement. Traditional ultrasound tomography methods often demand a large number of sensors, resulting in increased data processing and cost. Our model’s capacity to deliver high-resolution predictions with fewer sensors makes it an attractive option for industrial applications, where the primary goal is to detect single inclusions in homogeneous substrates.

While our research has showcased promising results, it is crucial to acknowledge its limitations. The current approach focuses on two-dimensional location predictions, limiting the model to estimating object positions within a reservoir cross-section. Future research may explore the extension of this approach to three-dimensional predictions, offering a more comprehensive spatial understanding.

Additionally, our dataset includes cases with a range of zero to four objects in the tank. Future work should expand this dataset to accommodate a broader spectrum of inclusion numbers and sizes. Determining the limits in terms of the maximum number of objects and the minimum detectable diameter will be an important avenue for future exploration.

This research has introduced a cutting-edge approach to object detection in the domain of ultrasound tomography. The demonstrated accuracy and reliability of our models hold great promise for a wide range of applications, particularly in fields where non-invasive imaging and precise object detection are imperative. While there are limitations to address and further research to be conducted, our work lays a strong foundation for advancements in ultrasound tomography and object detection using machine learning methodologies.

## Conclusion

This article has presented a novel approach to object detection in the field of ultrasound tomography. Feature extraction from a data frame consisting of three independent signals recorded by ultrasound sensors allows for the prediction of the number of objects inside the tank, their sizes, and positions due to an arbitrarily chosen system of coordinates. The data used in the article has not been significantly preprocessed, thus preserving the essential features of the apparatus and measurement systems. It has been shown that the model architecture based on two convolutional layers connected with dense layers allows for predictions with high accuracy. This approach is based on a relatively small number of sensors and is a prominent way of reducing the total cost of the final possible product.

This work has proposed a block-structured algorithm that separately solves classification and regression tasks. Both models are similar in their design, while they differ in the last layer and selected activation functions adapted to particular tasks. The article presents the metrics of the presented models, as well as examples of predictions obtained from the test data. The example of predictions in Fig 5 perfectly shows the high accuracy of the developed model.

It has been shown that using a dataset consisting of 24,000 records obtained from the presented measuring setup, it is possible to develop an algorithm for object detection. However, the proposed solution has its limitations. First, a cross-section through the reservoir is considered by which location prediction is made in only two dimensions. Second, the prepared learning set contains cases from zero to four objects in the tank. Such an approach could be extended for a larger number of inclusions with a wider range of their diameters. Determining a limit on the maximum number of objects and the minimum detectable diameter requires additional future research.

## References

[1] Tole NM, et al. Basic physics of ultrasonographic imaging. World Health Organization; 2005.

[2] Manbachi A, Cobbold RSC. Development and Application of Piezoelectric Materials for Ultrasound Generation and Detection. Ultrasound. 2011;19(4):187–196. doi: 10.1258/ult.2011.011027

[3] Pierce AD. Acoustics: an introduction to its physical principles and applications; 1992.

[4] Pierce AD. Wave equation for sound in fluids with unsteady inhomogeneous flow. The Journal of the Acoustical Society of America. 1990;87(6):2292–2299. doi: 10.1121/1.399073

[5] Duric N, Littrup P, Roy O, Schmidt S, Li C, Bey-Knight L, et al. Breast imaging with ultrasound tomography: Initial results with SoftVue. In: 2013 IEEE International Ultrasonics Symposium (IUS); 2013. p. 382–385.

[6] Tan C, Li X, Liu H, Dong F. An Ultrasonic Transmission/Reflection Tomography System for Industrial Multiphase Flow Imaging. IEEE Transactions on Industrial Electronics. 2019;66(12):9539–9548. doi: 10.1109/TIE.2019.2891455

[7] Majerek D, Rymarczyk T, Wójcik D, Kozłowski E, Rzemieniak M, Gudowski J, et al. Machine Learning and Deterministic Approach to the Reflective Ultrasound Tomography. Energies. 2021;14(22). doi: 10.3390/en14227549

[8] Soleimani M, Rymarczyk T. A 3D Lung imaging using ultrasound computed tomography; 2022.

[9] Jiang L, Wu B, Wei X, Lv X, Xue H, Lu G, et al. Flexible lead-free piezoelectric arrays for high-efficiency wireless ultrasonic energy transfer and communication. Mater Horiz. 2022;9:2180–2190. doi: 10.1039/D2MH00437B 35686946

[10] Soleimani M, Rymarczyk T. A Tactile Skin System for Touch Sensing with Ultrasound Tomography. Sensors. 2023;23(13). doi: 10.3390/s23136071 37447920 PMC10346191

[11] Mann R, Stanley SJ, Vlaev D, Wabo E, Primrose K. Augmented-reality visualization of fluid mixing in stirred chemical reactors using electrical resistance tomography. Journal of Electronic Imaging. 2001;10(3):620–629. doi: 10.1117/1.1379975

[12] Bolton GT, Primrose KM. An overview of electrical tomographic measurements in pharmaceutical and related application areas. AAPS PharmSciTech. 2005;6(2):E137–E143. doi: 10.1208/pt060221 16353970 PMC2750524

[13] Hiva Movafagh GT, Ein-Mozaffari F. Using tomography images to study the mixing of wheat straw slurries. Biofuels. 2016;7(4):365–375. doi: 10.1080/17597269.2015.1138038

[14] Gradov D, González G, Vauhkonen M, Laari A, Koiranen T. Experimental and Numerical Study of Multiphase Mixing Hydrodynamics in Batch Stirred Tank Applied to Ammoniacal Thiosulphate Leaching of Gold. Journal of Chemical Engineering’I&’ Process Technology. 2017;08. doi: 10.4172/2157-7048.1000348

[15] Rymarczyk T, Kłosowski G. Innovative methods of neural reconstruction for tomographic images in maintenance of tank industrial reactors. Eksploatacja i Niezawodność—Maintenance and Reliability. 2019;21(2):261–267. doi: 10.17531/ein.2019.2.10

[16] Jacques S, Pile K, Barnes P, Lai X, Roberts K, Williams R. An in-situ synchrotron X-ray diffraction tomography study of crystallization and preferred crystal orientation in a stirred reactor. Crystal Growth and Design. 2005;5(2):395–397. doi: 10.1021/cg0497288

[17] Germishuys Z, Manley M. X-ray micro-computed tomography evaluation of bubble structure of freeze-dried dough and foam properties of bread produced from roasted wheat flour. Innovative Food Science’I&’ Emerging Technologies. 2021;73:102766. doi: 10.1016/j.ifset.2021.102766

[18] Ricard F, Brechtelsbauer C, Xu XY, Lawrence CJ. Monitoring of Multiphase Pharmaceutical Processes Using Electrical Resistance Tomography. Chemical Engineering Research and Design. 2005;83(7):794–805. doi: 10.1205/cherd.04324

[19] Wajman R, Banasiak R, Mazurkiewicz L, Dyakowski T, Sankowski D. Spatial Imaging with 3D Capacitance Measurements. Measurement Science and Technology. 2006;17:2113. doi: 10.1088/0957-0233/17/8/009

[20] Koulountzios P, Aghajanian S, Rymarczyk T, Koiranen T, Soleimani M. An Ultrasound Tomography Method for Monitoring CO(2) Capture Process Involving Stirring and CaCO(3) Precipitation. Sensors (Basel). 2021;21(21). doi: 10.3390/s21216995 PMC8587525 34770301

[21] Wójcik D, Przysucha B, Gołabek M, Wośko E, Rymarczyk T, Adamkiewicz P. Image Reconstruction in Ultrasound Reflection Tomography using Quick High-Resolution Method. Journal of Physics: Conference Series. 2022;2408(1):012010.

[22] Baran B, Szyszka P, Majerek D. Ultrasound dataset created by Netrix S.A.; 2023. Available from: https://sync.netrix.com.pl/share.cgi?ssid=0LDC4wY.

[23] Zivkovic M, Bacanin N, Antonijevic M, Nikolic B, Kvascev G, Marjanovic M, et al. Hybrid CNN and XGBoost Model Tuned by Modified Arithmetic Optimization Algorithm for COVID-19 Early Diagnostics from X-ray Images. Electronics. 2022;11(22). doi: 10.3390/electronics11223798

[24] Sharmin S, Ahammad T, Talukder MA, Ghose P. A Hybrid Dependable Deep Feature Extraction and Ensemble-Based Machine Learning Approach for Breast Cancer Detection. IEEE Access. 2023;11:87694–87708. doi: 10.1109/ACCESS.2023.3304628

[25] Abadi M, Agarwal A, Barham P, Brevdo E, Chen Z, Citro C, et al. TensorFlow: Large-Scale Machine Learning on Heterogeneous Systems; 2015. Available from: https://www.tensorflow.org/.

[26] Soppin S, Ramachandra M, Chandrashekar BN. Essentials of Deep Learning and AI: Experience Unsupervised Learning, Autoencoders, Feature Engineering, and Time Series Analysis with TensorFlow, Keras, and Scikit-Learn (English Edition). BPB Publications; 2021.

